# Middle-aged preparation for healthy aging: a qualitative study

**DOI:** 10.1186/s12889-022-12715-x

**Published:** 2022-02-10

**Authors:** Mahnaz Solhi, Razieh Pirouzeh, Nasibeh Zanjari

**Affiliations:** 1 Department of Education and Health Promotion, School of health, Iran University of Medical Sciences, Tehran, Iran; 2 Health Education and Health Promotion, School of health, Iran University of Medical Sciences, Tehran, Iran; 3 Department of Health and Social welfare, Iranian Research Center on Ageing, University of Social Welfare and Rehabilitation Sciences, Tehran, Iran

**Keywords:** Middle-aged, Aging, Health, Preparation

## Abstract

**Background:**

Preparing for aging is investing resources in preparing to cope with the challenges that are expected to happen in older age. This will lead to an increase in the quality and well-being in aging. The present study was conducted to elucidate the preparation of the middle-aged people for healthy older age in Tehran, Iran.

**Methods:**

The present study was performed with a qualitative approach and using semi-structured interviews with 23 Iranian middle-aged people (45 to 59 years old), selected by purposive sampling, and the interviews continued until theoretical saturation. Data were analyzed using the content analysis technique with MAXQDA _10_ software.

**Results:**

The preparation of the middle-aged people was examined in four main categories including staying healthy, financial perspective for the future, outlook on aging, and planning for use time productively. The middle-aged people paid more attention to Staying healthy than other dimensions and the saw their future financially as very dark. Outlook on aging and planning for use time productively dimensions were less considered by the subjects.

**Discussion:**

Preparing for healthy aging is a multidimensional concept. The results of the study showed that the middle-aged studied in Iranian society rarely had the necessary preparation and planning to have healthy aging, also preparation is outside the individual behavioral factors and is more affected by the economic situation of the society. So, interventions and the country’s macro-policy are needed to plan for healthy aging these people.

## Background

Life expectancy is continuously increasing in both developing and developed countries [1, 2]. The old people make up 12% of the world’s population, and that number is predicted to be doubled by 2050 and tripled by 2100 [3].

Iran’s population is also currently in the process of transitioning the age structure from youth to old age. The United Nations Population Division predicts that, based on the assumed average growth rate during the 2040s and 2050s, about 25% of Iran’s total population will be in the age group of over 60, which is about a quarter of Iran’s population in the coming decades [4]. Today, the term “healthy ageing” is widely used defined as a lifelong process for optimizing opportunities to improve and maintain health, physical, social, and psychological well-being, independence, quality of life, and increasing the transition to a successful life [5].

At each stage of human development and growth is affected by the previous stage and affects the next stage, so the quality of life in old age is affected by the lifestyle of an adult and preparation for old age during adulthood should be built and established [6]. Middle age is a bridge between youth and aging. This period of life is the largest vital part of adulthood due to increased life expectancy. This stage of life is the most golden and fertile period of life [7, 8].

Part of the logical reasons for encouraging planning for old is that many people in the middle of life are not prepared to face the challenges of the next period of their lives and are vulnerable. Middle age is seen as an axial stage in life in which change can have a positive effect on the future [9, 10]. Preparing for aging is investing resources in preparing to face the challenges that are expected to happen at older ages. This can lead to increased quality of life and well-being in aging [11, 12].

Preparation is a concept that is investigated in many studies as preparation for retirement, while retirement is part of aging. These studies deal exclusively with preparing for events and challenges that happen directly and indirectly as you exit the job market [13–17].

Preparing for change in old age Instead of preparing for retirement can be a more comprehensive solution. Preparing for age-related change is not limited to old age itself, but should be a constant concern for the individual throughout life. Middle-aged adults and even younger ones should be involved in preparing for the expected changes in the future. However, as the distance from old age decreases, the preparation becomes more urgent and as a result more time is spent on it [18]. Most studies on preparation for old age are related to period after the entry aging [19–22]. For example Rattanamongkolgul’s study, prepared for aging including attention to physical health, spiritual health and readiness for death, and the elderly were actively preparing [21].

A limited number of studies investigated preparation for aging before reaching old age. For example In the study by Apouy et al. (2018), Half of the people said they were preparing for old age in financial, housing, social life and health [23].

Considering the importance of preparing for healthy aging and the lack of evidence-based research on middle-aged preparation for healthy aging in Iran, conducting qualitative research and explaining the perspective and status of middle-aged preparation for healthy aging can give us an understanding of the complexity of preparing for healthy aging. Increase and help build knowledge for possible interventions for future healthy aging policies and programs. Therefore, the aim of this study was to explain the Iranian middle-aged preparation for healthy aging.

## Methods

### Study design and participants

This qualitative study (as part of a larger project) was conducted in Tehran—the capital of Iran—via a conventional content analysis approach.

In total 23 middle-aged people were selected with purposeful sampling and the maximum variation based on their age, gender, level of education, job, marital status, place of residence, socio-economic, and cultural status. Inclusion criteria include being under 60 years of age and over 45 years of age and individuals who could express their thoughts and desire to participate in the study. Exclusion criteria included factors such as cognitive impairment, profound hearing loss, and speech problems that could prevent the interview.

### Procedures

Data was collected over a four-month period (January–April) in 2020 using semi-structured interviews consisting of three main questions. A sample interview guide was formulated in consultation with a qualitative researcher (NZ). Open-ended and guided questions were designed in order to encourage participants’ open communication of their preparation for healthy aging. Based on the participants’ answers, targeted probing questions were asked to further clarify or explain participants’ experiences in detail.

The three main questions were: “Are you prepared for aging?”, “What do you do to have a healthy aging period?”, and “Have you taken any steps to stay healthy in aging?

The duration of the interviews was between 35 and 115 min (with an average of 67 min).

### Analysis

A qualitative content analysis approach was used and the data collected were analyzed with MAXQD 10. The data analysis method used in this study was Schwandt, Lincoln & Guba (2007) [24]. The recorded interviews were first carefully listened to, and then, transcribed word-by-word and in detail. The semantic units including words, sentences, or paragraphs were determined in the text. Semantic units were combined based on similarity, and codes were extracted. Similar codes were grouped in subcategories, and then, the main categories were formed using an inductive process.

For data credibility, close interaction with participants, prolonged engagement in the field and notes in the field were used. To ensure data conformability, feedback, and defaults were checked to prevent their effect on data analysis and interpretation. The transferability of the results was increased through maximum diversity sampling, combining the study locations (health centers, parks, cultural centers and work place), and the selection of participants from different levels of education, age, gender, job, marital status, place of residence, socio-economic, and cultural status. With the help of similar results between the researcher and two experts involved in the data analysis process, the dependability of the research was achieved.

### Ethical considerations

This study was performed in accordance with the Declaration of Helsinki. Ethical approval was granted by the Research Ethics Committee at Iran University of Medical Sciences (Code: IR. IUMS. 1397.1043). Before enrollment in the study, enough explanations were provided to the participants about the purpose and method of the study, and written informed consent was taken from them. The participants were also assured about the confidentiality of the data and that they could leave the study whenever they wished.

## Results

In the present study, 11 women and 12 men were interviewed, the mean age was 50.43 ± 4.8 and the majority of the people (73.9%) were married (Table 1). Examples of middle-aged programs for healthy aging were categorized into four dimensions (Table 2).

**Table 1:** Demographic characteristics of the study sample (*N* = 23)

Characteristic	Frequency	Percentage
Gender		
Man Female	12 11	52 48
Marital status		
Married	17	73.9
Single	6	26.1
Education		
Under diploma	5	21.7
Diploma	8	34.78
Academic	10	43.47
Status of employment		
Employee	6	26.08
Worker	5	21.7
Housewife	5	21.7
Retired	2	8.69
Self-employment	5	21.7

**Table 2:** Main and sub-categories of middle**-**aged preparation for healthy aging

Categories	Sub-categories
Staying healthy	Promotes healthy habits of physical wellbeing Making mental health priority Improving Social Connectedness
Financial perspective for the future	Creating financial security Becoming a homeowner
Outlook on aging	Accepting the reality of aging Having positive view of aging
Planning for use time productively	Voluntarily activities Foresight for aging leisure

### Staying healthy

Keeping up health in middle age is an important factor in having healthy aging. Undoubtedly, those who have a healthier lifestyle will be better able to cope with the health changes in aging. The category of keeping up health was divided into two subcategories: Promotes healthy habits of physical wellbeing, making mental health priority, and Improving Social Connectedness.

#### Promotes healthy habits of physical wellbeing

From the perspective of the studied middle-aged people, promotes healthy habits of physical wellbeing was important for a healthy old age. Preventing declining health in old age through physical activity, eating healthy, screening, and periodic health examinations, quitting or starting to quit bad eating habits, quitting or starting to quit smoking, controlling diseases, Weight control, and eye examination expressed by participants. For example, many middle-aged people are eager to maintain healthy eating and talk about walking as physical activity.

“I eat fruits and vegetables; the main nutrition has always been in my diet and continues to be. I shouldn’t eat greasy, sweet and salty things.... I also have a lot of walking …” (45 years old man).

But most of the time, financial and time barriers were observed in this area so that some people hoped to improve their diet in aging because now their work affects their diet.

“Now we are out from morning to night and work, we have to eat whatever we can get...” (46 years old man).

In terms of health care, they were interested in the importance of their examinations and health check-ups, but they knew that this procedure is expensive: “I should go to the doctor right now. I may have a general illness, but I rarely go to the doctor. If you get sick, you have to spend money, you have a cold, you have to spend money, well, if a person is not financially secure, of course, this is not the case” (46-year-old woman).

#### Making mental health priority

Promoting mental health activities in middle age is important to have a good mental state in old age. The studied middle-aged people emphasized to live happily, to have a sense of peace in life, to strengthen positive thinking, to do pleasant things, to prevent depression, to avoid stress in life and anxiety to have a healthy old age for their future.

“Aging life, I lead a healthy life, of course, I do not go back to the past because grief overcomes me .... I strongly avoid negative people .... I try to stay away from negative thoughts, negative people, and negative places. For my comfort. I try to do things that I enjoy, I listen to music, I have guests and I have a mountain” (50 years old woman).

Participants of the low class of the society said they are not in a mentally good situation and blamed the economic situation of the society.

“The thought that it’s not good, will ruin it all.” A future that does not make you depressed... Most people are not depressed. A bad economic situation does not allow us to be mentally healthy. It is not clear what will happen in the future ...”. (45 years’ man).

#### Improving social connectedness

Social Connectedness is essential throughout a person’s lifespan. To prevent the feeling of separation and loneliness in the period of old age, one should think about expanding the circle of communication from the middle age. This subcategory includes concepts such as family communication, group orientation, having a companion, having good friends, and desire to go to a nursing home due to loneliness. A 55 years old woman is trying to build better family relationships in the family and is also thinking of expanding her relationships:

“I do not like to be old, to be alone...I try to improve our relationship with my husband and children, I don’t want to do anything to be alone; from now on I will start gathering those I lost like my friends and sisters. (55 years old woman)”.

### Financial perspective for the future

Financial perspective for the future**,** before entering aging, can meaningfully help well-being in aging. Perceived financial adequacy of individuals was included in two sub-categories of creating financial security and becoming a homeowner.

#### Creating financial security

Financial security in the middle-aged in this study included adequacy of savings, adequacy of pension rights, financial independence, adequacy of financial income, having financial management of savings, investment, insurance, financial independence, and job security. Men talked more about income generation and economic turmoil and the inability to save.

This concern was mostly seen in the workers. These people did not have job stability and security, and financial readiness for old age did not make sense for this group because they were struggling to make ends meet. A 46 years old worker says: “Saving cannot be done. You may only feed the self... I have no savings... Saving in our country! Well, inflation is unpredictable and uncontrollable.”

For women, financial independence was more important. A 46-year-old woman says: “Before I got old, I loved having money and I used to buy a house for myself and when I got tired, I would go and be alone for myself. For a woman, it better to be financially independent and does not need her husband.”

#### Becoming a homeowner

The environment and the place where a person live undoubtedly have a considerable effect on his/her health. In this study, buying housing and having personal housing, housing in the right place, inability to have housing, thinking about going to a nursing home due to lack of housing, having features such as elevators, and bathrooms were from the features of housing for the aging, which was mentioned by the subjects. But some were unable to provide or purchase housing for aging.

“Well, I would like to have a house and everyone wants to own a house, I tried to buy it but it didn’t work... I think I can no longer rent a very small house at all due to the expensive housing situation, finally, a person can rent a very small house ...” (45 years old woman).

### Outlook on aging

What is formed from aging and its concept in the minds of the middle-aged people will affect the quality of coping with it. Outlook on aging is told to two subcategories of accepting the reality of aging and positive view of aging.

#### Accepting the reality of aging

Avoiding the acceptance of aging can create challenges for people when dealing with aging. In this sub-category, not accepting aging, being involved in other issues, current problems, observing decreased physical ability, unplanned thinking, thinking with observing other aging people, talking to family and spouse about plans, study, and communication with the aging are mentioned. Some people have accepted the reality of aging:

“Anyway, this is a part of life. It’s inevitable. We come to it, whether we like it or not, whether we are prepared or not” (46 years old woman).

Most middle-aged people tried not to think about aging and the future. Some also wanted to think about it when they reached aging: “Now, I’d rather think about the present situation than the future. I’ll think about it whenever it comes. It’s too early for me to think about it now, ….” (45 years old man).

#### Having a positive view of aging

Having a positive view of aging provides a platform for experiencing a good and healthy aging period. Positive views include: achieving perfection, the usefulness of children, having grandchildren, remaining useful capacities, experience, experience transfer period, accountability, and having enough time. Few spoke positively about aging. It is worth noting that people with a positive view were men.

“Every period of a person’s life is kind of sweet. We indeed get older, lose some of the skills, but we can take advantage of our aging. In aging your children marry, you have grandchildren... they are kind of beautiful” (49 years old man).

But in this study, negative views such as disability period, fear of loneliness, fear of dependence, child marriage, and separation, reduction of social relations were observed more. Middle-aged women in the study spoke of their negative feelings about aging. One of the biggest negative attitudes toward aging was the fear of being dependent on others.

“I believe aging is so fearful, … I don’t want to be old and disabled or need anyone...” (46 years old woman).

### Planning for use time productively

How to use time in aging can be challenging. Managing daily time and planning how to spend time in old age can have a positive effect on health. Planning for use time productively was included in two subcategories of expansion of voluntarily activities and foresight for aging leisure.

#### Voluntary activities

Spending free time in old age to help others or improve society is good for both others and the individual. Having a volunteering program in middle age to spend useful time in old age will be effective for healthy aging. This section includes activities such as membership in social networks, social centers, membership in charity associations, assistance to nursing homes, guardianship of orphaned children, and some of the voluntary perceived barriers.

Few participants thought they had more free time in aging and could do voluntary works. Among them, a man with higher education states that: “One of the major issues that I have considered, and moving to help the community for money, not mobility, is the establishment of NGOs to help animals, homeless animals such as dogs, cats and other animals and birds that are oppressed or sick to reach them?”

##### Foresight for aging leisure

People in their aging will have more free time. Predicting the necessary arrangements for spending this period of life will help improve the quality of life for the better and healthier. Activities such as traveling, music, reading, enjoying nature, having fun with animals, having a garden, living in a quiet environment with spending more time with family and friends are mentioned. Few middle-aged people thought of side programs to spend time in aging. In the following, examples of people who, to some extent, had plans and thoughts for this are given.

“I try not to be like the others; they get old, sit in a corner, and wait for death to come; I try not to be like that and I like to get busy doing my daughter’s work and my son’s work” (45 years old man).

## Discussion

This study aimed to explain and discover the preparation of Iranian middle-aged people for healthy aging. The results showed that preparation for healthy aging is a multidimensional concept and staying healthy was more important than other aspects of middle-aged people and financial perspective for the future was the most effective dimension on other dimensions.

To staying healthy of the middle-aged, they were willing to adopt healthy eating behaviors. Studies have shown that a healthy diet, including eating fruits and vegetables, limiting the intake of fat, sugar, and sodium, is important for good health in aging [19–21, 23]. Most of the physical activity mentioned by the participants was walking, and less referred to public or specific sports. This could provide a strategy for promoting physical activity by encouraging walking, which can be adapted as a part of everyday life. Studies show that walking has a positive relationship with physical and functional well-being in aging [25, 26].

Mental health is an integral part of health and well-being. Among the middle-aged subjects, the lack of depression, anxiety, and happy living were the most important factors in mental health to achieve healthy aging. A review of studies also shows that psychological well-being plays an important role in healthy aging [27]. Older people are at risk for mental health problems, and paying attention to emotional needs is a way to prevent feelings of loneliness in aging [20]. Stress management [28] avoiding concerns and anxiety [21] is necessary to prepare for the challenges of aging.

### In improving social connectedness

Participants considered loneliness and isolation in aging to be a serious obstacle to healthy aging. This concern is more common in women, especially women who have few children and are worried about being alone in aging. In a study by Halaweh (2018), aging women were also concerned about living alone in the later stages of their lives and considered it an obstacle to good aging [29]. Studies show that living alone and low social participation are known to increase the risk of disability, fatigue, and more health problems in aging [30–32]. Having strong family relationships in aging to escape loneliness and receive support is considered as very important in the views of the middle-aged subject. Family support for the aging, especially their children, is an important element of successful aging in Eastern societies such as Iran [33]. Researchers suggest that comfortable aging in Eastern countries may be associated with family and social relationships that promote open-mindedness and tolerance. Conversely, in Western countries, such as the United States, activity, interaction, and vitality are likely to be associated with good aging [34].

In terms of financial perspective for the future, participants were less prepared. This lack was more pronounced in low-income people, especially workers. These people did not have hope for their financial future, people did not have a financial plan for the future, and they are involved in the material problems of their current life period.

Most studies have examined financial planning for old age that shown that most people do not have the financial preparation and planning for the future of old age [17, 23, 35, 36]. The effect of the financial dimension on other dimensions of health in aging is very evident and considerable in this study. Having financial well-being is considered a catalyst for health in aging in the society we study.

Developing countries are facing development problems and the elderly population is being ignored. Therefore, they do not have a plan for the future of the population and will face the problems and challenges of the elderly population. So, these countries are not ready to face the challenge of aging and this problem as a change in the social and economic system causes serious challenges and serious problems for the elderly [36]. Financial planning and health planning are both potentially important ways to prepare and promote better health in the middle-aged and aging. Financial planning is related to health planning [37].

In the current research, Iranian middle-aged people wanted to have an independent home. People who were early in the middle age could not afford to buy a house due to rising housing prices. In the Apouey study, preparedness for aging was less of a measure in terms of housing provision and adaptation [23]. The living environment is very important for healthy aging. Because there are places where older people spend the most time, and the characteristics of home, neighbors, and community help people avoid or prevent severe functional limitations, disability, or chronic illness. Due to the growth of the elderly population in recent decades, the quality and characteristics of houses and neighborhoods will be special determinants for healthy aging [38].

In terms of Outlook on aging to have a healthy aging, middle-aged people did not like to think about aging, and some knew it early and some denied their increased age. Few of them accepted and thought about a few realities of aging. Contrary to the results of the present study, in the study of Yeung et al. (2013), almost all people were engaged in anti-aging activities and were psychologically prepared to accept old age. Instead of denying aging, people should understand what is happening and it is inevitable that if they are upset about the limitations of life, they will suffer from emotional illnesses and consequently will have less healthy aging.

Men had a more positive view than women. Middle-aged women in the study, having a negative view, clearly expressed their fear of disability and dependence on others in old age. Studies in other cultures show that adults and the elderly have a great value for their independence and independence [39–41]. Positive attitudes are associated with higher levels of satisfaction and lower levels of anxiety and depression in aging [42]. Yeung’s (2013) study showed that psychological planning for retirement is associated with a positive attitude toward retirement and psychological health [39]. Knowing and overcoming negative stereotypes about aging through behavioral interventions among the young and the middle-aged before entering aging will help healthy aging.

Regarding the planning for use time productively, participation in voluntary social activities was rarely included in the programs among the middle-aged in this study. Studies show that participation in volunteer activities in leisure time has positive effects on the health, happiness, and self-esteem of the aging. This type of social participation is a protective factor against social isolation and decreased physical activity in aging [40].

Foresight for aging leisure was less of a concern for the middle-aged subjects. Leisure activities have a great influence on vitality [41]. Leisure activities have a significant effect on vitality (51). Many studies have generally emphasized the importance of planning for daily activities and living arrangements in aging and retirement [19, 43, 44]. The middle-aged in Iranian society under study have been involved in work activities and economic problems and difficulties that have had a dark view on how-to live-in aging.

### Limitations of the study

There are some limitations associated with this study. The sick and hospitalized middle-aged people did not enter the study and the study population we studied was healthy middle-aged. The study was also conducted in the metropolitan area while the preparation of the middle-aged in rural areas or small towns may be different. It is suggested that future researches provide a better understanding of preparation for healthy aging by considering all the middle-aged and in different areas. Also, quantitative studies, taking into account the large and varied sample size, can pave the way for appropriate interventions and planning.

## Conclusion

The results of the study showed that the middle-aged studied in Iranian society rarely had the necessary preparation and planning to have healthy aging. According to the middle-aged, preparation is outside the individual behavioral factors and is more affected by the economic situation of the society. Considering the findings of the study, there are major barriers on the path of planning and preparation of the middle-aged for healthy aging most of which are related to macro policies and planners of the society is in the social welfare of citizens. The middle-aged need to think about the future and aging from now on, and this requires creating opportunities and situations in society for employment, education, recreational activities, and social participation. Healthy aging can be achieved if people are given opportunities for comprehensive health as a source of support.

## Data Availability

The datasets generated and analyzed during the current study are not publicly available due lack of consent of sharing raw material, but parts of the material can be available from the corresponding author upon reasonable request.

## Abbreviations

WHO: World Health Organization
IUMS: Iran University of Medical Sciences
